# Quantitative and Comparative Investigation of Plasmalogen Species in Daily Foodstuffs

**DOI:** 10.3390/foods10010124

**Published:** 2021-01-08

**Authors:** Yue Wu, Zhen Chen, Jiaping Jia, Hitoshi Chiba, Shu-Ping Hui

**Affiliations:** 1 Faculty of Health Sciences, Hokkaido University, Kita-12, Nishi-5, Kita-ku, Sapporo 060-0812, Japan; wuyue123@hs.hokudai.ac.jp (Y.W.); chenzhen@hs.hokudai.ac.jp (Z.C.); jiaping.jia.s1@elms.hokudai.ac.jp (J.J.); 2 Department of Nutrition, Sapporo University of Health Sciences, Nakanuma Nishi-4-2-1-15, Higashi, Sapporo 007-0894, Japan; chiba-h@sapporo-hokeniryou-u.ac.jp

**Keywords:** ethanolamine plasmalogen, choline plasmalogen, livestock and poultry, seafood, fatty acyls, molecular species, LC-MS/MS

## Abstract

Plasmalogens are an animal-derived functional phospholipid increasingly known as a safe and effective nutritional ingredient, however, the quantitation and comparison of plasmalogen species in foods is limited. In the present work, determination methods for dietary plasmalogens using liquid chromatography-tandem mass spectroscopy under positive and negative ionization modes were compared. The negative-mode method, which showed better selectivity, sensitivity, and accuracy, was then applied in 14 kinds of livestock, poultry, and seafood samples. Livestock and poultry showed abundant total plasmalogen (530.83–944.94 nmol/g), higher than fish (46.08–399.75 nmol/g) and mollusk (10.00–384.76 nmol/g). While fish and mollusk samples expressed healthier fatty acyl composition, with higher eicosapentaenoyl and more beneficial n-6/n-3 ratio than the land animal meats, especially for squid and octopus, with eicosapentaenoyl of 98.4% and 94.5%, respectively. The correlations among plasmalogen species varied in different foodstuffs with distinguishing patterns, suggesting the customizable strategies for achieving targeted plasmalogen species. These findings not only provided fundamental comparison of plasmalogen among daily foodstuffs, but also contributed to extend the dietary plasmalogen sources for health food development.

## 1. Introduction

**Figure 1:**
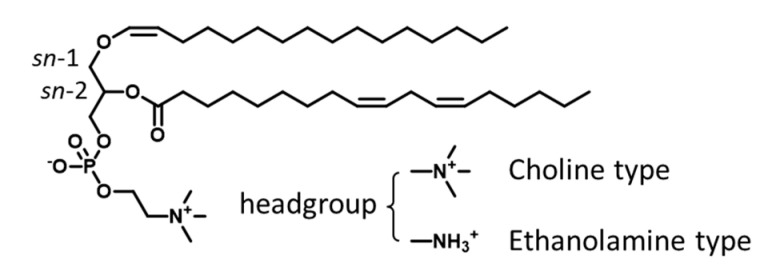
Structure of plasmalogen (taking choline plasmalogen p16:0/18:2n-6 as an example).

Plasmalogens are a kind of dietary phospholipid with beneficial health functions. It contains one fatty alcohol with a vinyl-ether bond at *sn*-1 position of the glycerol backbone, one fatty acyl chain at *sn*-2 position, and one phosphate headgroup linked to the glycerol backbone [1] (Figure 1). Plasmalogen participates in various physiological activities, including cell membrane maintenance and signal transduction [2]. A lack of plasmalogen can lead to Alzheimer’s disease, Parkinson’s disease, respiratory disease, and other lipid metabolic diseases [1,3,4,5,6]. Meanwhile, plasmalogen has been demonstrated to protect against oxidative damage [7,8], to inhibit inflammatory signals [9], and to suppress neuronal apoptosis [10,11]. Moreover, cumulative evidence suggested that food-derived plasmalogen could be considered as a potential therapeutic target for neurodegenerative and cardiometabolic disease [12]. Plasmalogen extracted from scallop and chicken have been reported to attenuate Alzheimer′s and Parkinson′s diseases [13,14], and regulate lipid metabolism [15] without showing adverse effect to health. Therefore, plasmalogens are increasingly known as a class of safe and effective food bioactive compounds.

The exploration of food bioactive compounds has been becoming very popular, as they possess protective effects in human body as well as promoting health and fitness [16]. Their food sources are the fundamental knowledge for therapeutic studies and health products development. With regards to plasmalogen, although it has been reported to exist in animals, such as livestock, poultry, and seafood [10,17], the quantitative comparation in different animal foodstuffs have not yet been well studied. To date, liquid chromatography-tandem mass spectrometry (LC-MS/MS) has been widely applied in the analysis of food products, such as bioactive constituent discovery, food authentication and discrimination, quality control, and so on [16,18,19,20]. For the source exploration of plasmalogen, most of the studies focused on ethanolamine plasmalogen (PlsEtn) [10,21], while the other major type, choline plasmalogen (PlsCho), attracted less attention. Although there have been reports on the composition of PlsEtn among different fishes and mollusks [17,22], the targeted differentiation between molecular species (not only the total carbons and total double bonds, but also the *sn*-1/*sn*-2 composition details), as well as the quantitative investigation of these plasmalogen, were quite limited.

Therefore, a highly selective and sensitive quantitation method for dietary plasmalogen species, which covers both PlsEtn and PlsCho, and clarifies fatty chain composition, is needed in food application. Herein, our study focused on the evaluation and comparison of plasmalogen species in common daily consumed foodstuffs, specifically, what kinds of foodstuffs contain more plasmalogens, which type of plasmalogen exist more in these foodstuffs, and how the fatty acyls compose in plasmalogen. The applicable LC-MS/MS parameters were confirmed, and the MS conditions under positive and negative modes were compared for the first time. Then, the typical livestock, poultry, fish, and mollusk foodstuffs were selected, and six representative plasmalogen species were simultaneously determined. Moreover, their plasmalogen variations were compared, and the nutritional values with regards to plasmalogen were discussed.

## 2. Materials and Methods

### 2.1. Chemicals

Spectral grade solvents for lipid extraction and LC/MS measurement, such as chloroform, isopropanol, methanol, and water, were purchased from Fisher Scientific (Pittsburgh, PA, USA). Ammonium acetate, isopropanol, and butylated hydroxytoluene (BHT) were obtained from Sigma-Aldrich (St. Louis, MO, USA). The plasmalogen standards, namely PlsEtn-p16:0/18:1n-9, PlsEtn-p16:0/18:2n-6, PlsEtn-p16:0/20:5n-3, PlsCho-p16:0/18:1n-9, PlsCho-p16:0/18:2n-6, PlsCho-p16:0/20:5n-3, and two internal standards (IS), PlsEtn-p16:0/17:0 and PlsCho-p16:0/17:0, which were commercially unavailable, were synthesized in our laboratory as previously reported [23]. Other chemicals were of HPLC grade and purchased from Kanto Chemical Co., Inc. (Tokyo, Japan) unless otherwise specified.

### 2.2. Sample Collection

In order to investigate the plasmalogen-enriched food sources, we selected the foodstuffs that are commonly used in our daily dishes. Four kinds of livestock and poultry meats (beef, pork, lamb, and chicken), five kinds of fishes (tuna, sea bream, salmon, flatfish, and amberjack), and five kinds of mollusks (shrimp, scallop, clam, octopus, and squid), were produced in Japan and were purchased from local markets during October to November 2019. The fresh samples were kept in ice, quickly transported to laboratory (within 20 min), and treated for analyze immediately. For the purpose of investigating commonly consumed foods in daily life, we selected the typical edible parts, such as chicken breast, pork leg, and fish lean meat. Their production area, part of the foodstuff, and sample numbers are listed in Table 1.

### 2.3. Lipid Extraction

Each batch of food sample was cut into small pieces prior to homogenization by an Abitelax^®^ kitchen blender (YOSHII ELECTRIC Co., Ltd., Gunma, Japan). Then, a proportion (approximately 50 mg) of the mashed sample was taken into a 1.5 mL Eppendorf^®^ Tube and weighed on an ultrasensitive electro-balance (Cubis^®^ ultra micro balance, Sartorius Inc., Göttingen, Germany). Thereafter, the sample was extracted twice with ice-cold chloroform/methanol 2:1 (with 0.2 nmol of each IS and 0.002% BHT) according to Folch′s method [24], followed by concentration under vacuum (CC-105 centrifugal concentrator, TOMY Seiko Co., Ltd., Tokyo, Japan). The yielded total lipid was dissolved in methanol and filtered to remove any residue prior to LC-MS/MS injection. The whole extraction procedure was performed within 1 h to avoid lipid auto-oxidation and degradation.

### 2.4. Chromatographic Separation

For chromatographic separation of plasmalogen species, an Accela pump system (Thermo Fisher Scientific Inc., Waltham, MA, USA) (coupled to a triple quadrupole MS) or a Prominence pump system (Shimadzu Corp., Kyoto, Japan) (coupled to an Orbitrap MS) was utilized. A Hypersil GOLD C8 column (1.9 µm, 50 mm × 2.1 mm, Thermo Fisher Scientific Inc., Waltham, MA, USA) was equipped and kept at 40 °C. The mobile phase consisted of methanol/water 5:1 *v*/*v* (with 10 mM ammonium acetate) (A) and methanol (B) at a flow rate of 200 µL/min, with the elution gradient shown in Figure S1. The tray temperature and the injection volume were set at 4 °C and 10 µL, respectively.

### 2.5. Quantification by Triple Quadrupole MS and MS/MS

A TSQ Quantum Access MAX mass spectrometer (Thermo-Fisher Scientific Inc., Waltham, MA, USA) with an electrospray ionization (ESI) probe was used, and both positive and negative ionization modes were investigated. The multiple reaction monitoring (MRM) via collision induced dissociation (CID) was applied for quantitation. The general hardware settings and the MRM parameters for all the analytes were optimized individually.

Quantitation was performed by internal standard method, and a series of diluted plasmalogen standard solutions were prepared for calibration curves, in which the peak area ratio of each plasmalogen to its corresponded IS (x) and the plasmalogen amount (y) was calculated. For sensitivity, the limits of detection (LOD) and quantification (LOQ) were defined as the signal-to-noise ratio (S/N) as 3 and 10, respectively. Repeatability was evaluated by six replicates of the intraday analysis and expressed as the coefficient of variation (CV) of the measurements. While accuracy was evaluated as the spike recovery at the 100%-spike-level from three replicates and calculated as follow:

Recovery% = (C_spiked sample_ − C_unspiked sample_)/C_added_ × 100%.

### 2.6. Identification by Orbitrap Tandem MS

An LTQ Orbitrap mass spectrometer (Thermo-Fisher Scientific Inc.) was used for identification of plasmalogen species with ESI under both positive and negative ionization modes. The capillary voltage was 3000 V, and the capillary temperature was 330 °C. The sheath gas (nitrogen) pressure and the auxiliary gas (nitrogen) were set to 50 units and 5 units, respectively. The MS^1^ data was obtained in Fourier Transform mode with resolving power 60,000 within the scan range *m*/*z* 600–900, while the MS^2^ and MS^3^ data were acquired using CID in ion-trap mode with the collision energy of 35 V and 40 V, respectively.

### 2.7. Data Process and Statistics

All the raw data were processed by the workstation Xcalibur 2.1 (Thermo-Fisher Scientific Inc.). The results were expressed as the mean ± standard deviation (SD). One-way ANOVA (using the Tukey′s post hoc test) was performed by Prism 8.0 (GraphPad Software, Inc., La Jolla, CA, USA), and *p* < 0.05 was considered to be statistically significant. Proportional stacked bar graph was made by using Excel 2019 (Microsoft Corporation, Redmond, WA, USA). Correlation analysis was conducted as the Pearson coefficients by using SPSS 26.0 (SPSS Inc., Chicago, IL, USA). And the heatmap with cluster dendrogram (Complete-linkage and Euclidean distance) was generated by R 4.0 [25] and pheatmap package [26].

## 3. Results and Discussion

### 3.1. Comparison of LC-MS/MS Methods under Positive and Negative Ionization Modes

The highly selective and instrument-friendly conditions are necessary for measuring plasmalogen in a variety of food samples. Therefore, in our study, each of the investigated plasmalogen species was optimized individually for LC-MS/MS general parameters under positive and negative modes (listed in Table S1), as well as the MRM parameters (listed in Table 2). Under our current optimized conditions in positive mode, the collisional activation of PlsEtn generated unique fragments of [glycerol + *sn*-2 chain]^+^ [27], while PlsCho was more likely to be fragmented to form the choline phosphate headgroup (*m*/*z* 184.0). In terms of negative mode, both PlsEtn and PlsCho showed the similar pattern of CID fragmentation, resulting in the [*sn*-2 fatty acyl]^−^ fragment. The fragmentation patterns are shown in Table 2. It should be noted that, different PlsCho species in positive mode showed the same product ion (*m*/*z* 184), which did not provide any side chain information of the molecules. Lipid components in food samples are complex and commonly exist as the mixture of isomers (the same total carbon and double bond numbers but different fatty acyl compositions). Consequently, we used the high-resolution Orbitrap MS and tandem MS to identify the possible plasmalogen isomers of our analytes, and compared with the results of the quantitative triple quadrupole MS/MS.

**Figure 2:**
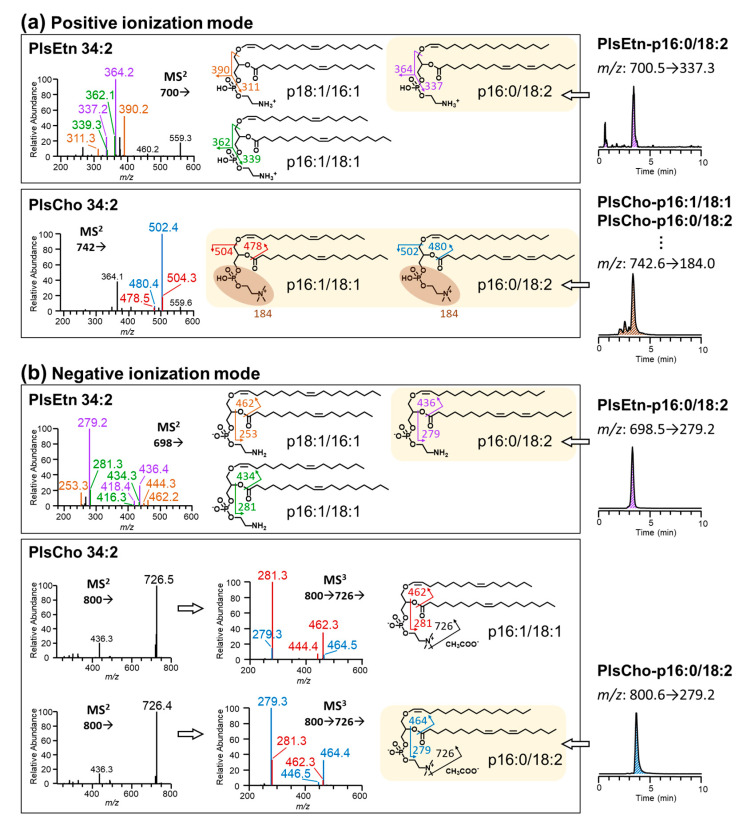
Identification for the isomer mixture of ethanolamine and choline plasmalogen in foodstuff samples by Orbitrap tandem MS, taking PlsEtn 34:2 (ethanolamine plasmalogen) and PlsCho 34:2 (choline plasmalogen) as examples. (**a**) Tandem MS fragmentation under positive ionization mode; (**b**) Tandem MS fragmentation under positive ionization mode. The structures indicate the existed isomers with different *sn*-1 and *sn*-2 fatty chains identified by MS^2^ and MS^3^. And the chromatograms on the right side were acquired by triple quadrupole MS/MS under the optimized MRM conditions for the analytes, respectively.

One tuna sample was chosen as the representative foodstuff sample, and the two representative plasmalogen species PlsEtn-p16:0/18:2n-6 and PlsCho-p16:0/18:2n-6 were investigated under both positive and negative modes with same chromatographic condition, individually (Figure 2). In foodstuff samples, PlsEtn 34:2 consisted of three major isomers, p18:1/16:1, p16:1/18:1, and p16:0/18:2, which shared the same MS^1^ spectrum ([M + H]^+^ *m*/*z* 700.5276 and [M − H]^−^ *m*/*z* 698.5130). MS^2^ under positive mode formed the diagnostic product ions (*m*/*z* 311.3, 339.3, or 337.2), which was consistent with previous report by Otoki et al. [28]. The same fragmentation was shown by triple quadrupole MS/MS (*m*/*z* 700.5 → 337.3 for PlsEtn-p16:0/18:2), while negative mode showed comparable diagnostic fragmentation of *sn*-2 fatty acyl, which was corresponded by triple quadrupole MS/MS (*m*/*z* 698.5 → 279.2 for PlsEtn-p16:0/18:2). Hence, both positive and negative modes could be used for selective detection of PlsEtn-p16:0/18:2n-6. In terms of PlsCho 34:2 ([M + H]^+^ *m*/*z* 742.5745 and [M + CH_3_COO]^−^ *m*/*z* 800.5811), Orbitrap tandem MS distinguished two isomers—p16:1/18:1 and p16:0/18:2—according to the primary fragmentation at the *sn*-2 position. However, these two isomers could not be discriminated by triple quadrupole MS/MS in positive mode: both of them (and even more isomers) fit the *m*/*z* 742.6 → 184.0, and thus, would be detected without selection. Even after optimizing MS/MS parameters, the intensities of *sn*-1/*sn*-2-diagnostic fragments (*m*/*z* 478/504 or 480/502) were still too weak to be selected as the sensitive diagnostic product ions, which was similar to other reports [28]. While in negative mode, triple quadrupole MS/MS showed distinctive fragmentation at *sn*-2 position (*m*/*z* 800.6 → 279.2). Therefore, it is considered that plasmalogen quantitation by triple quadrupole MS/MS under ESI negative mode might lead to a higher selectivity and specificity.

Moreover, we compared the method validation of these two conditions. The calibration curves under both positive and negative modes showed satisfied R^2^ (≥0.994 for all). And the repeatability as intraday CV were less than 5% for all species under both modes, except for PlsEtn-p16:0/18:1 under positive mode. The accuracy was also comparable (ranged from 92.6–111.0% for negative mode, and 78.0–106.4% for positive mode) (Table S2). While in terms of sensitivity, negative mode also showed lower LOD (≤6.1 pmol/g for all) and lower LOQ (≤9.2 pmol/g for all) than positive mode (LOD ≤ 32.6 pmol/g for all; LOQ ≤ 48.9 pmol/g for all) (Table S2). These results suggested us that the condition under negative mode might be better suitable for investigation of foodstuffs, because the variety of samples might show quite distinctive content of plasmalogen. To solve the problem in the selectivity and sensitivity of plasmalogen quantitation, Otoki et al. modified the LC-MS/MS condition under ESI positive mode by using alkali metal (e.g., lithium and sodium) additives in the mobile phase, which achieved the diagnostic ions of [C_3_H_4_OCH=CHR_1_]^+^ and providing high intensities [29], however, the induced nonvolatile ions might cause contamination and ion suppression in the ESI probe [30]. Based on our results, the LC-MS/MS method in negative mode was considered selective, sensitive, and instrument-friendly for analyzing large batches of foodstuff samples.

### 3.2. Total Plasmalogen Amount

**Figure 3:**
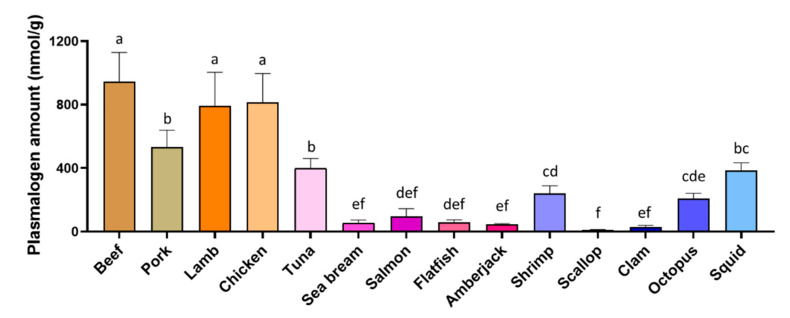
Comparison of total plasmalogen amount in foodstuffs. a–f, bars with different letters differ at *p* < 0.05, calculated by one-way ANOVA with the Tukey′s post hoc test. Data were expressed as means ± SD.

The amount of each plasmalogen species was determined in the 14 kinds of foodstuffs (Table 3). The total amount of plasmalogen, calculated as the sum of all the tested molecular species, varied distinctively among different foodstuffs (Figure 3). The total plasmalogen in livestock and poultry (530.83–944.94 nmol/g) were more than in fish (46.08–399.75 nmol/g) and in mollusk (10.00–384.76 nmol/g), while within livestock, pork (530.83 ± 109.00 nmol/g) showed less plasmalogen than other two meats (944.94 ± 183.50 nmol/g in beef, 792.61 ± 210.76 nmol/g in lamb). Among the five investigated fishes, tuna (399.75 ± 60.90 nmol/g) showed remarkably higher plasmalogen amount than others, and even accounted for approximately 4 folds of the second enriched fish salmon (95.53 ± 48.91 nmol/g). Regarding the mollusks, squid contained the highest plasmalogen (384.76 ± 48.29 nmol/g), followed by shrimp (239.77 ± 48.39 nmol/g) and octopus (208.88 ± 32.25 nmol/g), whereas scallop was the lowest (10.00 ± 3.13 nmol/g).

According to these data, we found out that land animals (especially beef, lamb, and chicken) contained more plasmalogen than seafoods. One of the possible explanations should be that water content is higher in fresh seafoods than land animals [31,32]. In our current study, following the common practice in daily life, all the samples were treated in fresh and calculated in wet weight, and the resulted data showed that within the same wet weight, livestock and poultry were hopeful to provide more abundant plasmalogen. Therefore, they were likely to be the more favorable sources for the development of plasmalogen-enriched health products.

### 3.3. Types of Plasmalogen (Phosphate Headgroup)

**Figure 4:**
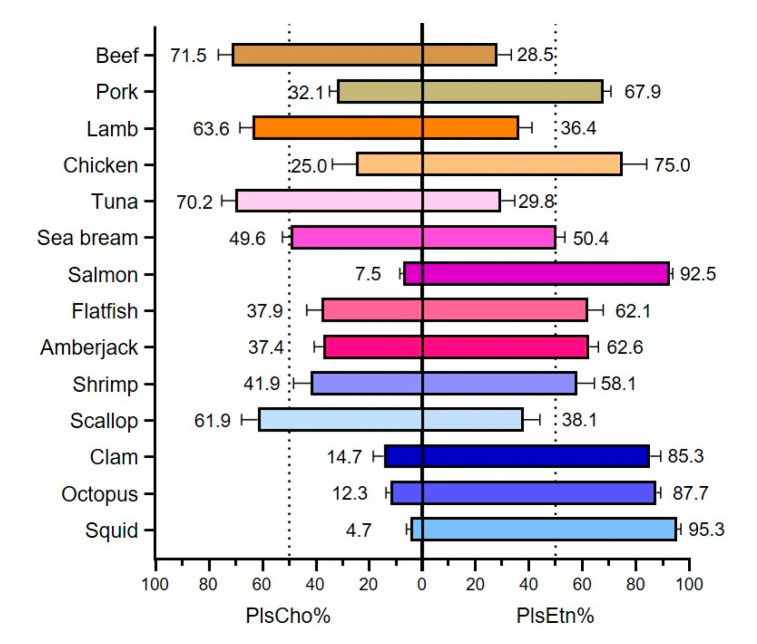
Proportion of ethanolamine and choline as the plasmalogen headgroup in foodstuffs. Data were normalized as percentages and shown as means ± SD.

We also compared the phosphate headgroup among the tested samples in our study (Figure 4), in which 11 out of 14 kinds of foodstuffs showed greater proportion of PlsEtn (exceed 50%), especially for squid, salmon, octopus, and clam (accounting for 95.3%, 92.5%, 87.7%, and 85.3%, respectively). While PlsCho was the major type in beef, tuna, lamb, and scallop, with the proportion of 71.5%, 70.2%, 63.6%, 61.9%, respectively. However, we did not find the unique pattern of phosphate headgroup among these foodstuffs. Most of the previous studies focused on PlsEtn of its clinical significance or beneficial effects [10,17]. But in our recent study, besides PlsEtn, PlsCho also exhibited a protective function on human hepatocytes and attenuated oxidative damages [8]. Though the differences between PlsEtn and PlsCho of their bioactive potentials have not been elucidated, our current findings might provide a fundamental basis of plasmalogen type (headgroup) composition in food sources.

### 3.4. Fatty Acyl Composition of Plasmalogen

**Figure 5:**
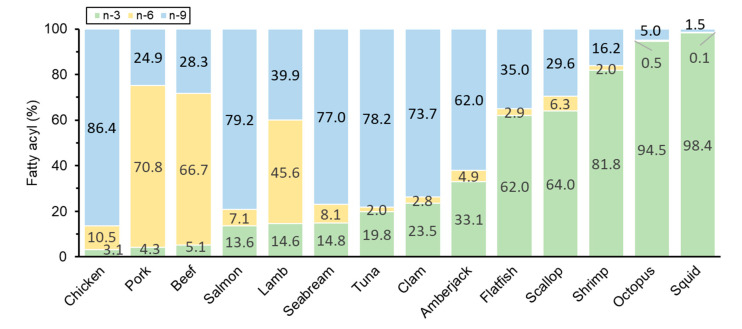
Fatty acyl composition of plasmalogen in foodstuffs. Data were normalized as percentages, and bars of foodstuffs were sorted by the increasing percentage of the n-3 fatty acyl eicosapentaenoyl.

Fatty acids play an important role in dietary and have been known as one of the evaluating factors for nutritional value, therefore, in this study, the fatty acyl composition in plasmalogen from the investigated foodstuffs was compared. The results showed differences among animal sources (Figure 5). Overall, plasmalogen from land animal meats contained more linoleoyl, while seafoods showed higher eicosapentaenoyl. It is known that marine animals contain more n-3 fatty acyls [33], and the present results of plasmalogen also support this viewpoint. According to WHO and European Nutritional Societies, a lower n-6/n-3 ratio (typically less than 10 or 5) was recommended for prevention of inflammatory, cardiovascular, and neurological disorders [34,35]. While in our study, the plasmalogen-derived n-6/n-3 ratio in marine foods ranged from less than 0.1 in squid to 0.6 in sea bream (3.1–16.6 in land animals as comparison). Although marine products showed less plasmalogen amount, they expressed healthier fatty acyl composition with regards to n6/n3 ratio, suggested more nutritional value as well as benefit to cardiovascular health.

In the land animals, chicken showed more oleoyl (86.4%) as the n-9 fatty acyl and less eicosapentaenoyl (3.1%) than livestock. While within livestock, pork and beef showed similar fatty acyl composition, which were higher in n-6 fatty acyl and lower in n-3 and n-9 fatty acyls than lamb. These results were consistent of Fogerty et al. that in PlsEtn the oleoyl ratio: chicken > lamb > beef ≈ pork, and the eicosapentaenoyl ratio: pork > beef > lamb > chicken [36]. Our study indicated that it is necessary to select suitable animals to obtain in large quantities of plasmalogen with favored fatty acyl in breeding and food industry. In the seafoods, all the foodstuffs shared the same characteristics of low linoleoyl ratio (less than 10% for all) but differed between fishes and mollusks for oleoyl and eicosapentaenoyl ratio. Oleoyl was more abundant in fishes, accounting for 79.2% in salmon, 77.0% in seabream, 78.2% in tuna, and 62.0% in amberjack, whereas eicosapentaenoyl was more enriched in mollusks, especially for squid and octopus, reaching up to 98.4% and 94.5%, respectively.

It was noticed that scallop showed a relatively lower eicosapentaenoyl ratio of 64.0% in these mollusks. Some studies used scallop-derived plasmalogen as n-3 fatty acyl-enriched fraction for bioactivity evaluation [9,13], in which the plasmalogen sources might come from the whole internal parts (not only muscle but also viscera). But in our study, we only selected the adductor muscle as the popular edible part. It has been reported that different parts of mollusk provided lipid variance [37,38]. Therefore, it is hinted that the more detailed plasmalogen profile among different parts (and even for inedible parts) of foodstuffs should be studied individually in the future. Another factor for plasmalogen variation within the same marine foodstuff is the environment, such as geographic location, season, temperature, and so on [39,40]. In the current work, most of the marine foodstuffs were produced in Hokkaido area of Japan (about 43° N 142° E) within 2 months, which limited the environment-induced variance towards the samples. Still, it is worth investigating the plasmalogen variations of the same foodstuff to better develop the favorable plasmalogen sources.

### 3.5. Correlations among Different Plasmalogen Species in These Foodstuffs

**Figure 6:**
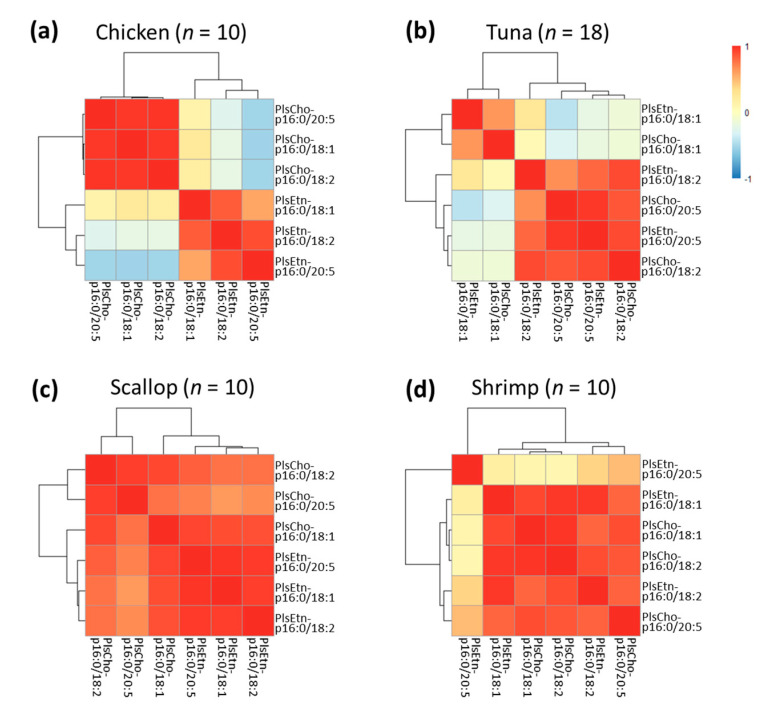
Correlation of plasmalogen species in chicken (**a**), tuna (**b**), scallop (**c**), and shrimp (**d**), calculated as Pearson coefficients. Variables with a high positive correlation to each other were expressed in red, while negative correlations were indicated in blue.

The correlation analysis was performed by calculating the Pearson coefficient of each two plasmalogen species within the same foodstuff, summarized as heatmap (The typical patterns are shown in Figure 6, and the others are shown in Figure S2). Chicken showed a clear correlation characteristic that there were strong positive correlations both among PlsEtn species and among PlsCho (coefficients = 0.850–0.971, *p* < 0.01 for all; except for PlsEtn-oleic/PlsEtn-EPA as coefficient of 0.573), but not between PlsEtn and PlsCho (Figure 6a). This result indicated a competitive accumulation of PlsCho and PlsEtn in chicken. Therefore, it might be promising to promote the targeted type of plasmalogen (either PlsCho or PlsEtn) enriched in chicken by customized feeding in modern agriculture. A similar situation was observed in tuna, in which strong positive correlation was seen between the two oleic-contained species and among the polyunsaturated fatty acyl (PUFA)-contained species (coefficients = 0.634–0.962, *p* < 0.01 for all), but not between oleic- and PUFA-contained species, suggesting a selective production of fatty acyls in plasmalogen (Figure 6b). Thus, it might also be possible to achieve the favorable fatty acyl composition in plasmalogen from tuna. In terms of scallop, all the species were highly correlated (coefficients = 0.714–0.980, *p* < 0.05 for all), indicating the non-competitive accumulation for all plasmalogen species (Figure 6c). This pattern was also revealed in beef, lamb, sea bream, and salmon (Figure S2a,c–e), while in shrimp, most of the species were similarly correlated as in scallop, though PlsEtn-p16:0/20:5n-3 was the only one that kept independent with the others (|coefficients| < 0.5 for all) (Figure 6d). The conservative variety of this species hinted that shrimp might be a stable source of dietary PlsEtn-p16:0/20:5n-3. Although the common biosynthetic routine of plasmalogen has been reported [41], whether there are specific pathways of plasmalogen accumulation in these animal foodstuffs remained uncovered. Nevertheless, the current study prompted that, by elucidating the biosynthetic pathways, it might be possible to develop the improved feeding or breeding of plasmalogen-enriched animals, to extend the sources of dietary plasmalogen.

## 4. Conclusions

In summary, the current study optimized and compared the quantitative methods for six representative plasmalogen species by LC-MS/MS, and applied the optimum method to livestock, poultry, fish, and mollusk foodstuff samples. The total plasmalogen amount were higher in livestock and poultry than seafoods, while seafoods contained more beneficial n-6/n-3 fatty acyl composition, especially for mollusks. These comparative results might provide a better understanding towards the potential plasmalogen-enriched foodstuffs. Moreover, the correlations among these plasmalogen species were found with distinguishing patterns in different foodstuffs, which suggested a potential strategy to promote achieving the targeted plasmalogen species in food production. Our present work might contribute for further studies on developing the extended plasmalogen functional food sources. In the future, it should be necessary to compare the biological activities of the specific plasmalogen constituents from different foodstuffs.

## Figures and Tables

**Table 1:** Information of the investigated foodstuff samples.

Type	Foodstuff	Part	Sample Numbers (n)	Production Area
Livestock & Poultry	Beef	Leg	12	Kamikawa, Hokkaido (43°50’ N 142°21’ E)
	Pork	Leg	10	Tokachi, Hokkaido (42°54’ N 143°22’ E)
	Lamb	Leg	6	Tokachi, Hokkaido (43°05’ N 142°51’ E)
	Chicken	Breast	10	Iburi, Hokkaido (42°33’ N 141°22’ E) (4); Okhotsk, Hokkaido (44°11’ N 143°05’ E) (6)
Fish	Tuna	Lean meat	18	Oshima, Hokkaido (41°43’ N 141°01’ E) (14); Nagasaki, Kyushu (33°27’ N 129°45’ E) (4)
	Sea bream	Lean meat	10	Nemuro, Hokkaido (43°20’ N 145°35’ E) (6); Kisarazu, Chiba (35°22’ N 139°53’ E) (4)
	Salmon	Lean meat	10	Kushiro, Hokkaido (43°03’ N 144°51’ E) (6); Minamisannriku, Miyagi (38°43’ N 141°34’ E) (4)
	Flatfish	Lean meat	4	Kushiro, Hokkaido (43°03’ N 144°51’ E)
	Amberjack	Lean meat	6	Shiribeshi, Hokkaido (43°18′ N 140°36′ E)
Mollusk	Shrimp	Muscle	10	Nemuro, Hokkaido (43°20’ N 145°35’ E)
	Scallop	Muscle	10	Nemuro, Hokkaido (43°20’ N 145°35’ E)
	Clam	Muscle	16	Kushiro, Hokkaido (43°03’ N 144°51’ E)
	Octopus	Arms	4	Oshima, Hokkaido (41°43’ N 141°01’ E)
	Squid	Mantle	4	Oshima, Hokkaido (41°43’ N 141°01’ E)

**Table 2:** MRM parameters in positive and negative modes.

Ionization Mode	Molecular Species	Precursor Ion	Fragmentation	MRM Transition	Collision Energy	Tube Lens
Positive	PlsEtn-p16:0/18:1	[M + H]^+^	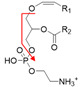	702.5 → 339.3	20	115
	PlsEtn-p16:0/18:2	[M + H]^+^		700.5 → 337.3	20	110
	PlsEtn-p16:0/20:5	[M + H]^+^		722.5 → 359.3	17	115
	PlsEtn-p16:0/17:0	[M + H]^+^		690.5 → 327.3	20	120
	PlsCho-p16:0/18:1	[M + H]^+^	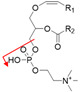	744.6 → 184.0	25	126
	PlsCho-p16:0/18:2	[M + H]^+^		742.6 → 184.0	25	110
	PlsCho-p16:0/20:5	[M + H]^+^		764.6 → 184.0	25	120
	PlsCho-p16:0/17:0	[M + H]^+^		732.6 → 184.0	25	110
Negative	PlsEtn-p16:0/18:1	[M − H]^−^	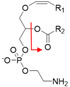	700.5 → 281.2	33	134
	PlsEtn-p16:0/18:2	[M − H]^−^		698.5 → 279.2	33	149
	PlsEtn-p16:0/20:5	[M − H]^−^		720.5 → 301.2	28	134
	PlsEtn-p16:0/17:0	[M − H]^−^		688.5 → 269.2	33	143
	PlsCho-p16:0/18:1	[M + CH_3_COO]^−^	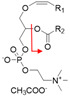	802.6 → 281.2	40	149
	PlsCho-p16:0/18:2	[M + CH_3_COO]^−^		800.6 → 279.2	41	115
	PlsCho-p16:0/20:5	[M + CH_3_COO]^−^		822.6 → 301.2	31	111
	PlsCho-p16:0/17:0	[M + CH_3_COO]^−^		790.6 → 269.2	37	125

PlsEtn: ethanolamine plasmalogen; PlsCho: choline plasmalogen.

**Table 3:** Amount of each plasmalogen species in foodstuffs (nmol/g, means ± SD).

Foodstuff	PlsEtn-p16:0/18:1	PlsEtn-p16:0/18:2	PlsEtn-p16:0/20:5	PlsCho-p16:0/18:1	PlsCho-p16:0/18:2	PlsCho-p16:0/20:5
Beef	66.11 ± 11.79	179.21 ± 65.08	30.78 ± 22.12	201.15 ± 22.13	450.74 ± 70.24	16.95 ± 10.70
Pork	94.42 ± 12.34	242.70 ± 60.50	20.98 ± 6.94	37.77 ± 6.51	133.27 ± 47.15	1.70 ± 0.24
Lamb	86.92 ± 23.75	121.58 ± 48.15	81.23 ± 31.59	229.07 ± 52.29	239.46 ± 67.45	34.36 ± 11.27
Chicken	533.23 ± 125.27	54.41 ± 12.21	19.83 ± 6.86	171.04 ± 78.37	31.22 ± 14.23	5.04 ± 2.69
Tuna	87.95 ± 31.05	2.37 ± 1.13	30.11 ± 18.47	224.52 ± 23.61	5.76 ± 2.87	49.03 ± 33.97
Sea bream	20.20 ± 8.08	2.38 ± 1.14	5.39 ± 2.10	21.86 ± 6.07	2.05 ± 0.74	2.71 ± 0.96
Salmon	72.69 ± 39.28	6.45 ± 3.04	9.72 ± 4.40	3.01 ± 1.07	0.37 ± 0.10	3.29 ± 1.31
Flatfish	16.35 ± 6.84	1.13 ± 0.22	18.67 ± 6.67	3.65 ± 1.16	0.53 ± 0.15	16.73 ± 2.40
Amberjack	16.91 ± 3.64	1.41 ± 0.32	10.61 ± 1.40	11.67 ± 1.05	0.85 ± 0.18	4.63 ± 0.83
Shrimp	14.17 ± 2.63	1.66 ± 0.30	121.93 ± 22.72	24.71 ± 6.98	3.17 ± 1.04	74.13 ± 24.98
Scallop	1.14 ± 0.52	0.10 ± 0.04	2.65 ± 1.03	1.82 ± 0.59	0.54 ± 0.16	3.75 ± 1.05
Clam	19.94 ± 9.50	0.58 ± 0.18	4.01 ± 1.39	1.00 ± 0.30	0.22 ± 0.09	2.68 ± 0.55
Octopus	1.62 ± 0.11	0.21 ± 0.03	181.48 ± 29.25	8.84 ± 1.52	0.90 ± 0.19	15.83 ± 2.51
Squid	0.79 ± 0.09	0.11 ± 0.02	366.16 ± 50.43	5.03 ± 0.26	0.30 ± 0.02	12.37 ± 3.89

## Data Availability

Data available on request.

## Supplementary Materials

The following are available online at https://www.mdpi.com/2304-8158/10/1/124/s1, Table S1: MS general hardware settings, Table S2: Comparison of the method validation between positive and negative modes, Figure S1. Eluting gradient for chromatographic separation of plasmalogen species, Figure S2: Correlation of plasmalogen species in different foodstuffs, calculated as Pearson coefficients.
