# Supplementary material for: Quantitative and Comparative Investigation of Plasmalogen Species in Daily Foodstuffs
Source: Foods. 2021 Jan 8;10(1):124. doi: 10.3390/foods10010124 (PMC7827193; doi:10.3390/foods10010124)
Supplement: Supplementary file 1 [file foods-10-00124-s001.pdf]

Supporting information

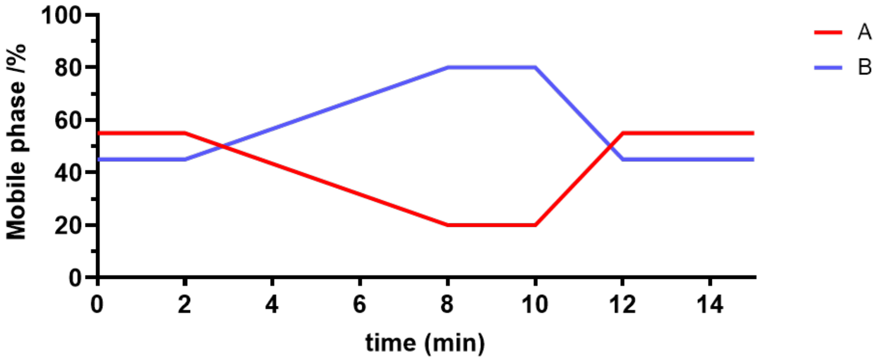

Time /min	0	2	8	10	12	15
A /%	55	55	20	20	55	55
B /%	45	45	80	80	45	45

Figure S1. Eluting gradient for chromatographic separation of plasmalogen species.

Table S1. MS general hardware settings

Ionization mode	Ion spray voltage	Vaporizer temperature	Sheath gas pressure	Aux gas pressure	Capillary temperature	Collision pressure
Positive	4000 V	500 °C	20 psi	35 psi	300 °C	1.5 mTorr
Negative	3750 V	250 °C	10 psi	20 psi	370 °C	1.6 mTorr

Table S2. Comparison of the method validation between positive and negative modes

Mode	Molecular species	Linearity		Sensitivity		Repeatability	Accuracy
		Equation	R^2	LOD (pmol/g)	LOQ (pmol/g)	Intraday CV (%)	Recovery (%)
Positive	PlsCho-p16:0/18:1	y=10.455x+1.979	0.9967	15.7	23.5	3.0	100.4 ± 6.1
	PlsCho-p16:0/18:2	y=11.692x+2.128	0.9985	13.4	20.1	2.3	78.0 ± 4.6
	PlsCho-p16:0/20:5	y=15.241x+1.739	0.9980	8.4	12.6	3.3	88.4 ± 6.3
	PlsEtn-p16:0/18:1	y=14.014x−0.113	0.9960	10.7	16.1	7.8	90.7 ± 6.4
	PlsEtn-p16:0/18:2	y= 4.852x+0.300	0.9950	10.7	16.0	3.6	106.4 ± 6.7
	PlsEtn-p16:0/20:5	y=21.508x−0.230	0.9949	32.6	48.9	3.9	79.9 ± 5.6
Negative	PlsCho-p16:0/18:1	y=24.063x−4.254	0.9992	6.1	9.2	3.0	101.1 ± 4.1
	PlsCho-p16:0/18:2	y=22.904x−4.250	0.9991	5.2	7.8	4.2	105.7 ± 3.3
	PlsCho-p16:0/20:5	y=71.648x−3.035	0.9982	3.3	4.9	3.5	100.5 ± 1.2
	PlsEtn-p16:0/18:1	y=11.848x+1.606	0.9992	4.2	6.3	2.9	94.4 ± 3.8
	PlsEtn-p16:0/18:2	y= 9.355x−1.103	0.9965	4.2	6.3	2.7	92.6 ± 4.1
	PlsEtn-p16:0/20:5	y=27.087x−0.471	0.9969	2.6	3.8	2.1	111.0 ± 2.9

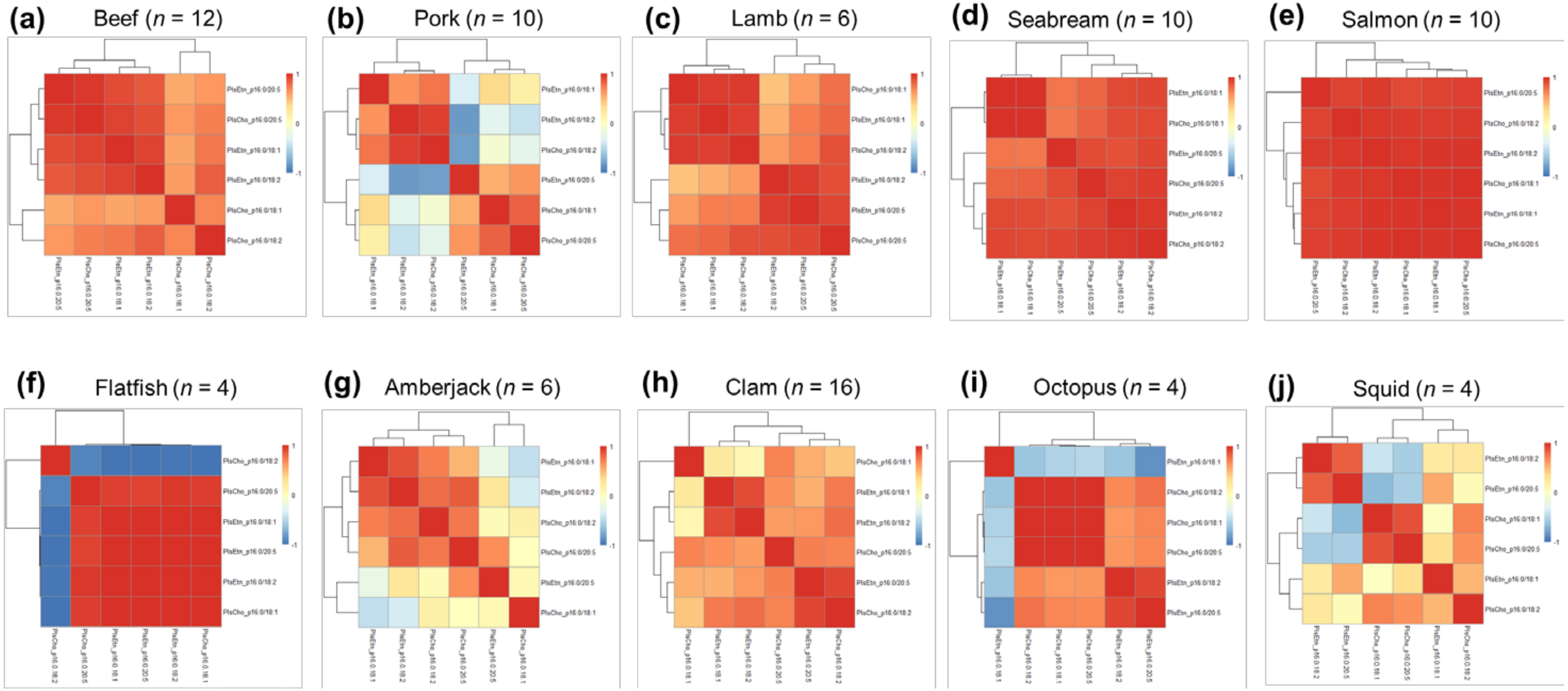

Figure S2. Correlation of plasmalogen species in different foodstuffs, calculated as Pearson coefficients. Variables with a high positive correlation to each other were expressed in red, while negative correlations were indicated in blue.
